# Seasonal Changes in the Genetic Structure of an Aphid-Ant Mutualism as Revealed Using Microsatellite Analysis of the Aphid *Tuberculatus quercicola* and the Ant *Formica yessensis*

**DOI:** 10.1673/031.009.0901

**Published:** 2009-03-27

**Authors:** Izumi Yao, Shin-Ichi Akimoto

**Affiliations:** ^1^ 21st COE Program (Neo Science of Natural History), Graduate School of Science, Hokkaido University, Sapporo 060-0810, Japan; ^2^ Department of Ecology and Systematics, Graduate School of Agriculture, Hokkaido University, Sapporo 060-8589, Japan

**Keywords:** microgeographic, genetic differentiation, tree-dwelling aphid, clonal mixing, supercolony, *Quercus dentata*

## Abstract

The present study examined whether the mutualistic relationship between the aphid *Tuberculatus quercicola* (Matsumura) (Homoptera: Aphididae) and the attending ant *Formica yessensis* Forel (Hymenoptera: Formicidae) has had any mutual effects on the microgeographical genetic population structure of both partner species. The aphids and the attending ants were collected in June, August, and October 2004 from six trees of the Daimyo oak *Quercus dentata* Thunberg (Fagales: Fagaceae) and were genotyped using microsatellite loci. Significant genetic differentiation was detected among *T. quercicola* populations on the respective trees across seasons (an average of pairwise *F*_ST_ = 0.183). Similarly, significant genetic differentiation was found among populations of *F. yessensis* that attended aphid colonies on the respective host trees, though the averages of pairwise *F*_ST_ were lower (an average of pairwise *F*_ST_ = 0.070). An analysis of molecular variance and two-way ANOVA detected a significantly large genetic difference between spring and summer samples in *F. yessensis* but not in *T. quercicola*, indicating that changes in genetic composition occurred in the *F. yessensis* colony. In spite of a drastic seasonal change in the genetic difference in *F. yessensis*, principle coordinate analysis showed that the relative position among the six populations was maintained from spring to summer, suggesting that the tree where honeydew was available for a long time was occupied by *F. yessensis* over the same period and that the honeydew sources were inherited at the level of the ant colony. It is hypothesized that the suitability of host trees for the aphid *T. quercicola* may have an affect on the genetic structure of the attending ant *F. yessensis*. Within a colony of aphids, clonal diversity decreased significantly as the season progressed. The reduction in clonal diversity may be due to an increase in identical genotypes by parthenogenesis or selective pressure from host plant deterioration.

## Introduction

Mutualisms are reciprocal interactions in which one partner performs some beneficial services to its associate and receives some reward from the associate (Bronstein 1994). When mutualistic associations are found in a community, the foundation of populations of one partner species can be highly influenced by those of the associated species. However, the initiation and maintenance of mutualism are not only affected by the interactions between the partners, such as distribution or genetic variation among species, but also by the physical environments. These factors acting on both partners determine the distribution of mutualism in a community and whether the mutualism is constant or ceases to exist in the course of time. Recently, attempts to understand the genetic interaction between organisms in association with their genetic structure have provided a framework for ‘community genetics’, which integrates population genetics and community ecology (Antonovics 1992; Neuhauser et al. 2003; Whitham et al. 2003).

Mutualism between aphids and ants has been widely studied in evolutionary ecology (Way 1963; Stadler and Dixon 2005) and may be a good model for understanding community genetics. Aphids feed on phloem sap circulating in the vascular system of a plant and excrete honeydew, a liquid waste-product rich in sugar, but poor in amino acids. Honeydew plays a critical role in the mutualistic interactions between ants and aphids. Attending ants collect honeydew directly from aphids. The aphids, in return, benefit from the protection offered by the ants against natural enemies and the fungal pathogens that grow on their excretion.

The Daimyo oak, *Quercus dentata* Thunberg (Fagales: Fagaceae), was a dominant tree species in the sampling location, which was a grassy open site running parallel to the seashore and acting as the transition zone from sandy coast to oak forest. In late spring, larval fundatrices of the aphid *T. quercicola* hatch on the trunk from over-wintered eggs and move to the underside of developing leaves. *T. quercicola* does not alternate host plants during its life history. During the summer, all nymphs of the aphid develop into alate viviparous females, which produce offspring parthenogenetically. In autumn, alate males and apterous oviparous females appear. After mating, oviparous females move from the leaves to the branches to deposit eggs. The ant *F. yessensis* is known to have super colonies comprising thousands of nests containing about 360 million workers and over a million queens along the Ishikari coast, Hokkaido, northern Japan (43°N, 141°E) (Ito 1973). The annual cycle of colony activities and development of nest structure with some environmental phenology were reported by Ito (1973). Extra-nest activities by post-hibernating workers begin in mid-April just after thaw. Full-scale activity starts in late May when honeydew becomes available. Budding of new ant colonies occurs generally from May to July. New sexuals, produced in a limited number of nests, emerge in late July to early August and leave the nest for nuptial flights in early August. New workers emerge slightly after the sexuals in late July to mid-September. Extra-nest activities drop in mid-September to early October and virtually cease in November. In accordance with the observation by Ito (1973), the number of ants attending aphid colonies increase until late June, followed by a decline by late July. Thereafter, the number of ants in aphid colonies sharply increases in abundance again (Yao unpublished observations). Experimentally excluding ants from aphid colonies always resulted in extinction within a month, indicating that the aphids strongly depend on the ants for protection against natural enemies (Yao et al. 2000).

Such a mutualism, however, does not always occur since not all *Q. dentata* trees are suitable for aphids and not all aphid colonies last until autumn. These are mainly due to the sensitivity and specificity of aphids for host plants. Thus, it can be hypothesized that the formation and maintenance of this mutualism is affected by the difference in susceptibility of host plants for aphids, differences in predation pressures among host plants, and the seasonal changes in host plant quality (Yao 2004). *Q. dentata* individuals that can support many aphid colonies throughout seasons would be important resources, not only for aphids, but also for the attending ants. If honeydew produced by *T. quercicola* residing on certain trees is an available resource across all seasons, locating a colony near the tree would be advantageous for *F. yessensis*. Therefore, the interaction with aphids is inherited at the ant colony level. If a honeydew-foraging area is inherited by an ant colony from season to season, genetic similarity should be detected between old and new *F. yessensis* workers across seasons on a single host tree. To test this hypothesis, it is most appropriate to genotype individuals using high resolution microsatellite markers because it is a co-dominant marker and can detect a large number of alleles along with a simple pattern of Mendelian inheritance.

To understand the broader significance of community evolution, it is necessary to show that, under natural conditions, selection acts on genetic differences at a community level (Whitham et al. 2003). Since viviparous aphid females propagate parthenogenetically over the summer, most aphids within a colony are assumed to belong to a clonal group. Recently, several studies using genetic markers have revealed that a number of different clones were contained within a single aphid colony (Setzer 1980; Abbot et al. 2001; Johnson et al. 2002), which points to the possibility that uneven fitness occurs through altruistic behavior within an aphid colony. Therefore, estimating the clonal diversity within a single aphid colony will provide helpful information in understanding the evolution of multi-level selection. In order to determine the genetic structure of aphid-ant mutualism, especially the degree of clonal mixing within a single aphid colony, a single sampling is insufficient because seasonal deterioration in host plant quality also affects the maintenance of the mutualism. As almost all aphid species are entirely dependent on the phloem sap, seasonal deterioration in host plant quality is a crucial factor affecting their survival and reproduction (Awmack and Leather 2002). The concentrations of nitrogen and carbohydrates in phloem sap are high in spring and autumn when leaves are growing or senescent and low in summer when leaves are mature (Dixon 1970). Through seasonal deterioration in phloem sap, plants may reallocate nutrition to a limited number of parts, resulting in a resource variation within the same plant. This within-tree variation may be responsible for dispersal of aphids and changes in the genotypic diversity within aphid colonies.

In the present study, we examined the genetic structure of both partners in the aphid-ant mutualism, focusing on the following three specific questions: (1) what is the extent of genetic differentiation among host trees colonized by aphids for both species, (2) whether genetic similarity on a single host tree for both species is maintained between two consecutive seasons, and (3) whether the level of clonal mixing within *T. quercicola* colonies change seasonally.

## Materials and Methods

### Study area and studied species

**Figure 1.**
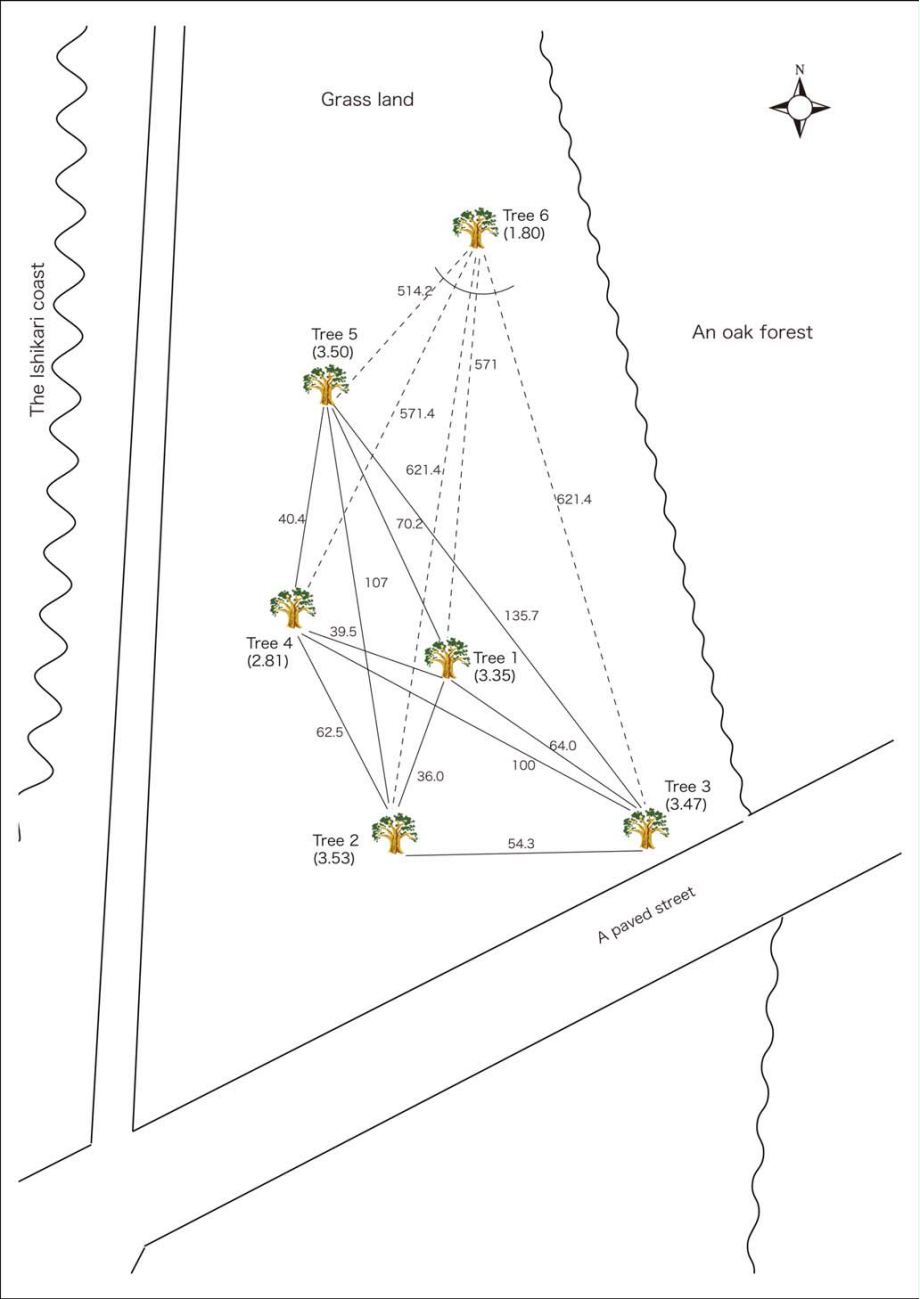
Distribution of trees used in the present study. Tree height (m) is given in parentheses. Distances among trees (m) are given as an actual measurement (solid line) and estimated measurement (broken line) of aerial photos.

The aphids and ants were collected on dunes on the Ishikari Coast. The dunes were covered by the dominant species *Miscanthus sinensis* (Hitchc.) Ohwi, *Rosa rugosa* Thunberg, *Celastrus orbiculatus* Thunberg, and *Carex kobomugi* Ohwi. Although many *Q. dentata* were distributed widely throughout the study area, a few oak trees on which the mutualism between *T. quercicola* and *F. yessensis* lasts until autumn have been observed over nine consecutive years from 1995 to 2003. Such trees are never found in the adjacent oak forest, perhaps because *F. yessensis* needs an open site for nest development. The nests near an oak tree are recognizable because their surface has mounds consisting of decayed stalks, blades and hulls of *M. sinensis*. Six trees (on average 3.1m tall), on which the mutualism occurred in every year were selected for the monitoring of the seasonal changes in the genetic structures of aphids and ants on each tree. With this monitoring, it is possible to determine whether the initial differences in the population structures between host trees are maintained across seasons. The location of each tree is shown in Figure 1.

### Collection of aphids and ants

A preliminary study showed that the aphid population grew exponentially until August, followed by a steep decline by late September. Thereafter, the population increased in density again (Ito and Higashi 1991; Yao 2004). Aggregations of *T. quercicola* along the midrib of the upper or lower surface of a single leaf of *Q. dentata* were defined as a colony. The number of aphids and attending ants per aphid colony were counted before collection (*N* in Table 1). Collection was conducted three times: (1) 1st to 4th June 2004, hereafter referred to as spring samples and corresponding to population foundation by the first generation, (2) 31st July 2004, hereafter referred to as summer samples, and (3) 12th October 2004, hereafter referred to as autumn samples, which consisted of the oviparae and winged males. Four to eight aphids and two to five attending ants were collected from a single aphid colony. Two to eight colonies of each of the six trees were sampled three times through the seasons in 2004. The sample sizes of the three subsets were as follows: for the spring sample, a total of 33 colonies averaging 5.4 ± 0.7 SD aphids and 3.8 ± 1.0 SD ants (corresponding to 69% and 78% of the total number of aphids and ants per aphid colony, respectively); for the summer sample, a total of 43 colonies averaging 7.1 ± 1.0 SD aphids and 4.3 ± 0.6 SD ants (corresponding to 33.3% and 56.4% of the total number of aphids and ants per aphid colony, respectively); for the autumn sample, a total of 32 colonies averaging 7.7 ± 0.8 SD aphids and 2.6 ± 0.8 SD ants (corresponding to 43.9% and 78.7% of the total number of aphids and ants per aphid colony, respectively). No colonies were found in two trees (trees 5 and 6) in the autumn, probably due to the deterioration in host plant quality. All aphid colonies collected are listed in Table 1. The aphids and attending ants collected were stored in acetone (Fukatsu 1999) and held at -20 °C prior to the experiment.

### Microsatellite analysis

To extract DNA, the whole aphid or hind femur of an ant was used. Genomic DNA was extracted following the Chelex procedure (Walsh et al. 1991), and resuspended in 15 µ″l of TE (10 mM Tris-HCl, 1 mM EDTA, pH 8). Five microsatellite loci were used to examine the genotypes of individual *T. quercicola* (Tq15, Tq17, Tq18, Tq23, and Tq26; Yao et al. 2003) and *F. yessensis* (Fy3, Fy4, Fy7, and Fy15; Hasegawa and Imai 2004, and FL12; Chapuisat 1996). Because many stutter bands emerged in some *T. quercicola* individuals amplified by Tq23, the design of the forward primer was changed to 5′-TCACACGCGCATACGATATT-3′. Forward primers were labeled with Beckman Dye fluorescence (Proligo). The polymerase chain reaction (PCR) amplifications and determination of allele sizes for both species were the same as described in Yao et al. (2003) and Hasegawa and Imai (2004).

### Analysis of genetic data

Individuals with a failure in PCR amplifications, four for *T. quercicola* and 10 for *F. yessensis*, were excluded from all analyses. Because clonal reproduction in aphid populations probably leads to deviations from the Hardy-Weinberg equilibrium and linkage disequilibrium, all analyses except for clonal mixing analysis were carried out using only one aphid per genotype (Sunnucks et al. 1997). Only a few individuals with an identical genotype were detected in the ant populations; this allowed us to use the complete dataset of ants for genetic analyses.

The number of alleles, allele frequencies, and heterozygosity for the populations of the aphids and ants were calculated using the Microsatellite analyzer (MSA) 4.00 (Dieringer and Schötterer 2002). The gene diversity (*h*) of each microsatellite locus was calculated using the equation,

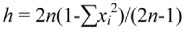

where *n* is the number of individuals examined and *x_i_* is the frequency of the *i*^th^ allele across all genotypes (Nei and Roychoudhury 1974).

Linkage disequilibrium between loci and departure from the Hardy-Weinberg equilibrium at each locus were tested using the Fisher's exact probability test with a sequential Bonferroni correction for multiple comparisons (Rice 1989) in Genepop (http://genepop.curtin.edu.au/) (Raymond and Rousset 1996).

### Differences in genetic structure between trees and between seasons

An analysis of molecular variance (AMOVA; Excoffier et al. 1992) was conducted using Arlequin version 2.0 (Schneider et al. 2000) to partition the genetic variation into components attributable to differences among the specified hierarchical groups (FCT), among populations within hierarchical groups (FSC), and within populations (FST). Three criteria were used to calculate the genetic variation. First, the genetic variation was calculated for among trees, among colonies within trees, and within aphid colonies. Second, the genetic variation was calculated among seasons, among trees within seasons, and within trees. Third, the genetic variation was calculated among seasons, among aphid colonies with seasons, and within aphid colonies. In addition to AMOVA, pairwise *F*_ST_ with a sequential Bonferroni correction for multiple comparisons was calculated using MSA 4.00 to examine the genetic differences among trees divided by seasons. Relationships between genetic differentiation and geographical distance separating trees (isolation by distance) were examined in each season using Isolde in Genepop. Isolation by distance was tested with a Mantel procedure (10 000 permutations) by correlating pairwise *F*_ST_ with the natural logarithm of the straight-line distance (m) between pairs of trees.

In order to examine whether the genetic differences among host-associated populations, if any, vary seasonally for both the aphids and ants, *F*_ST_ values were analyzed with two-way ANOVA model in which *F*_ST_ between two consecutive seasons on each tree was treated as the dependent variable, while seasons (spring-summer and summer-autumn) and trees were treated as independent variables. The interaction between trees and seasons failed to be included in the ANOVA because of the lack of data from Trees 5 and 6 in the autumn. The *F*_ST_ value was transformed to arcsine square-root in order to satisfy the requirement of normality. Furthermore, to examine the microgeographical and seasonal genetic similarities among populations on the six trees, principal coordinate analysis (PCO) (Gower 1966) with PCO 2.0 (Iwata 2005) was used.

Although the information that samples of both species was collected from six different trees and their genotypes were distinguished individually, which gives a measure of genetic diversity per tree, the actual extent of assemblage of individuals characterized by a set of allele frequencies at each locus across all trees or seasons is not clear. Thus, dividing the total sample into clusters of individuals, each of which fits some genetic criterion that defines it as a group, would provide an estimate of the number of biologically realistic subpopulations (Pearse and Crandall 2004). The potential existence of *K* subpopulations across the study area was addressed using the Bayesian clustering approach implemented in the software Structure 2.0 (http://pritch.bsd.uchicago.edu/software/structure2_beta.html) (Pritchard et al. 2000; Falush et al. 2003). We conducted two analyses to determine the effect of seasons on the subpopulations. First, the entire data set with the information on seasons not considered in the calculation was analyzed with assumed values of *K* ranging from one to 11 for *T. quercicola* and from one to six for *F. yessensis*. Second, the data set was analyzed each season separately with assumed values of *K* ranging from one to 10 for *T. quercicola* and from one to five for *F. yessensis*. As recommended in the manual for the software, an admixture model was chosen with the assumption that allele frequencies were independent in each population. For the selected model, the Bayesian posterior probabilities were calculated as an estimated Pr(*X*|*K*), where individual genotypes are assigned to a predefined number of clusters (*K*) in a given genotype (*X*). The program was run setting the length of the burn-in period and the number of MCMC replications after burn-in at 30,000 and 10^6^, respectively, and a maximum value of lnP(*X*|*K*) was obtained.

### Seasonal changes in clonal mixing in *T. quercicola* colonies

The numbers of genotypes of aphids per season per tree per colony and per tree were calculated using the Groups/Summary command in the statistical software package JMP 5.0.1 J (SAS 2002). The clonal diversity of *T. quercicola* in an aphid colony and within a tree were calculated using *s* = *G*/*n* (Llewellyn et al. 2003), where *G* is the number of different genotypes in an aphid colony and *n* is the number of individuals examined. Additionally, the Shannon-Wiener (S-W) diversity index (*H*) with the binary logarithm was used to determine clonal diversity as follows:

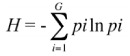

where *G* is the number of clones and *pi* the proportion of individuals in the sample that belong to clone *i*. The effects of seasons and host trees on the clonal diversity in a colony (*s* and *H*) were tested using a two-way ANOVA with JMP 5.0.1 J. The *G*/*n* ratio was transformed to arcsine square-root in order to satisfy the requirement of normality. Welch statistics was used in testing ANOVA because of its robustness against violations of assumptions.

**Table 1.**
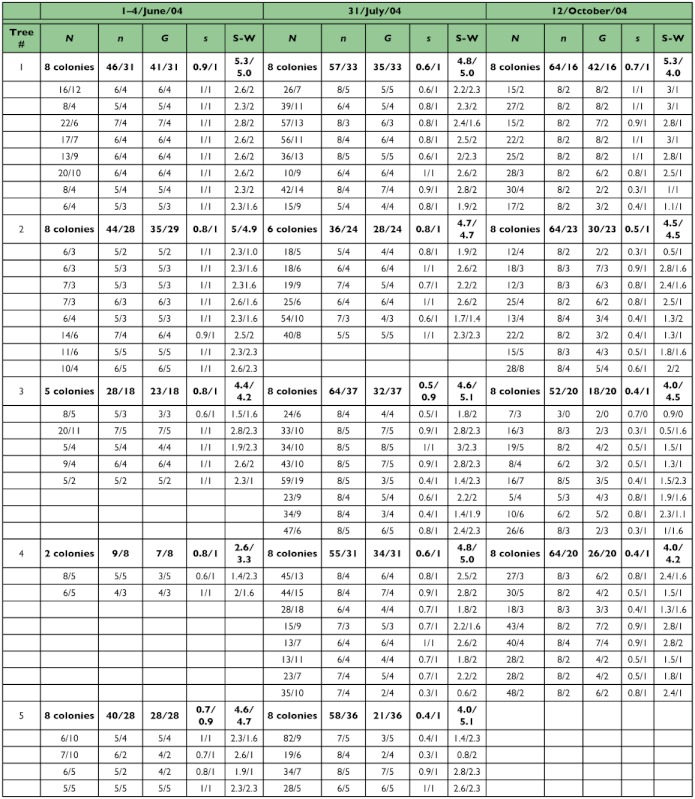
Collection data for the aphid *Tuberculatus quercicola* and the ant *Formica yessensis* from the oak tree *Quercus dentata*.

**cont:**
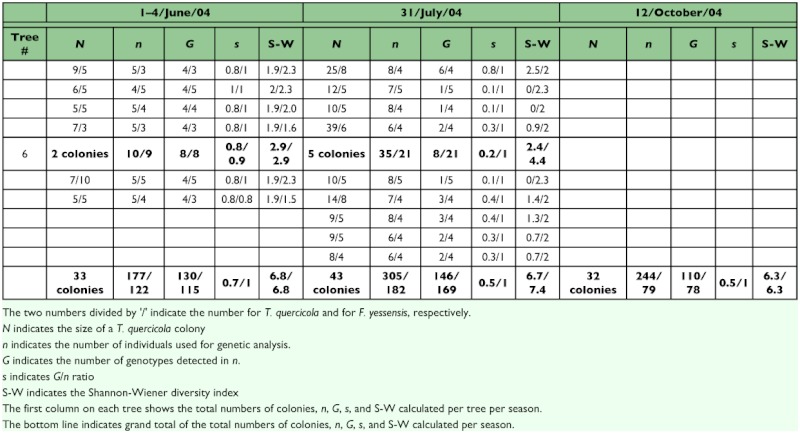

## Results

### Allelic variation and genotyping

The average number of allelic variants detected per locus for tree-associated populations of *T. quercicola* and *F. yessensis* ranged from two (Tq17 and Tq18) to 15 (Tq15), with a mean of 5.8 ± 4.79 SD and ranged from two (Fy4) to nine (Fy3 and Fy7), with a mean of 6.2 ± 2.79 SD, respectively. The gene diversity estimates for populations of *T. quercicola* and *F. yessensis* ranged from 0.40 (Tq18) to 0.73 (Tq23), with an average value of 0.55 ± 0.13 SD and ranged from 0.43 (Fy4) to 0.74 (Fy7), with an average value of 0.61 ± 0.12 SD, respectively. Only two loci (Tq15 and Tq23) had private alleles (an allele found in only one subpoplation) (Hartl and Clark 1997) with a frequency of more than 1% in each locus (Supplemental Data 1). Heterozygosity deviations from the Hardy-Weinberg equilibrium to heterozygosity excess for each locus across populations were significant at two loci of *T. quercicola* (Tq15 and Tq23) and *F. yessensis* (Fy7 and Fy15) (Fisher' s exact probability test; *P* < 0.0083 for both *T. quercicola* and *F. yessensis*) (Supplemental Data 2). Linkage disequilibrium was not detected for any pair of loci across all *T. quercicola* populations and *F. yessensis* populations with the Bonferroni correction for multiple comparisons.

The combined five microsatellite loci of *T. quercicola* and *F. yessensis* had a high resolution for discriminating different genotypes within a colony (Table 1). All but six colonies (94%) contained multiple clones of *T. quercicola* across seasons, and almost all *F. yessensis* workers visiting *T. quercicola* colonies had different genotypes (Table 1). 317 genotypes within 726 aphids and 356 genotypes within 383 ants were detected across seasons. Although common genotypes across the six trees were not found in populations of both species, a single genotype of *T. quercicola* and 19 genotypes of *F. yessensis* were found to be common in four trees and in several pairs of trees, respectively.

### Differences in genetic structure between trees and between seasons

For both of the populations of *T. quercicola* and *F. yessensis*, AMOVA indicated that significant levels of genetic partitioning was explained by trees (Table 2). Percentage of variation in analysis with partitioning the data among trees was higher in *T. quercicola* than in *F. yessensis*, which explained 10.5% and 4.1% of total genetic variation, respectively. Irrespective of setting the hierarchical subpopulation to trees or aphid colonies, partitioning the data among seasons provided a significant effect for *F. yessensis* but *T. quercicola*, indicating that season is the best predictor of genetic discontinuity for *F. yessensis*.

**Figure 2.**
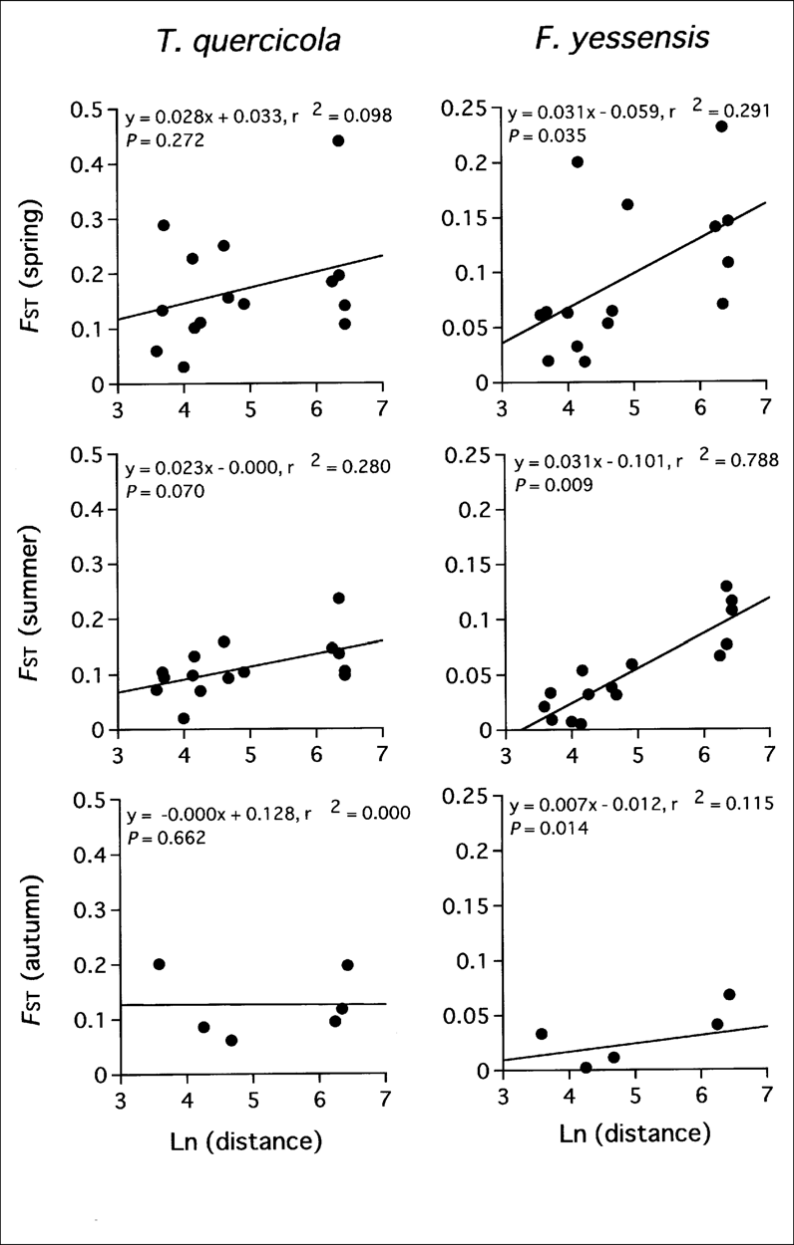
Analysis for isolation by distance for seasonal populations of *T. quercicola* and *F. yessensis*.

Pairwise comparisons of *F*_ST_ values between the tree-associated population in each season indicated that genetic differentiation among populations was greater in *T. quercicola* than in *F. yessensis* (the averages were as follows: for *T. quercicola*, for spring, 0.219 ± 0.127 SD, range 0.023–0.514, for summer, 0.159 ± 0.081 SD, range 0.020–0.329, for autumn, 0.151 ± 0.062 SD, range 0.067–0.201; for *F. yessensis*, for spring, 0.096 ± 0.067 SD, range 0.018–0.233, for summer, 0.053 ± 0.040 SD, range 0.004–0.129, for autumn, 0.045 ± 0.039 SD, range 0.002–0.108) (Table 3). Isolation by distance analysis showed that a significant positive relationship between *F*_ST_ and geographical distance (ln-transformed) was found for *F. yessensis* but not for *T. quercicola* (Figure 2).

**Table 2.**
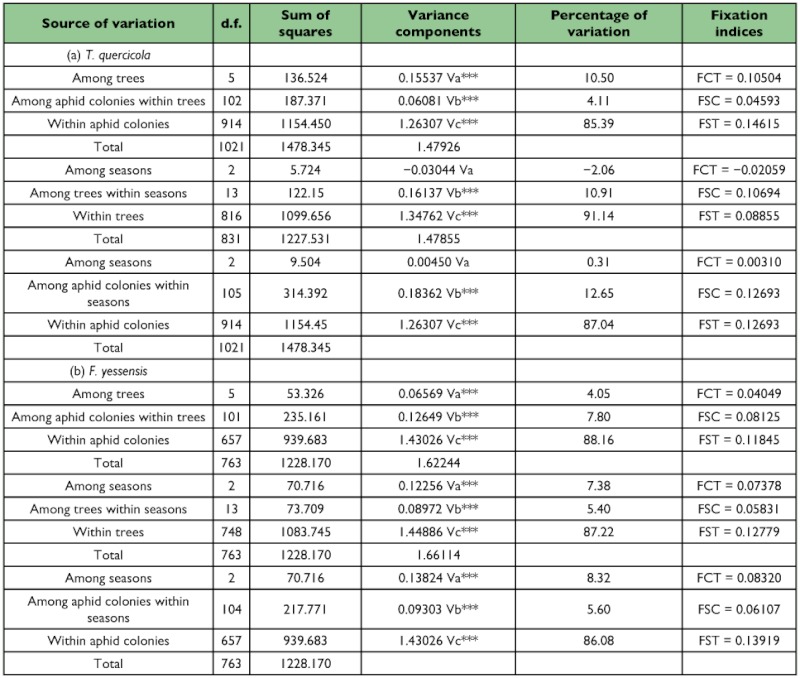
AMOVA results for populations of (a) *T. quercicola* and (b) *F. yessensis* partitioned by trees and seasons nested with trees or colonies.

A significant difference in the level of genetic differentiation between two consecutive seasons was found in *F. yessensis* but not in *T. quercicola* (Table 4). For *F. yessensis*, pairwise *F*_ST_ between spring and summer samples was greater than between summer and autumn samples (averaging 0.135 ± 0.006 SE between spring and summer, averaging 0.021 ± 0.008 SE between summer and autumn).

**Figure 3.**
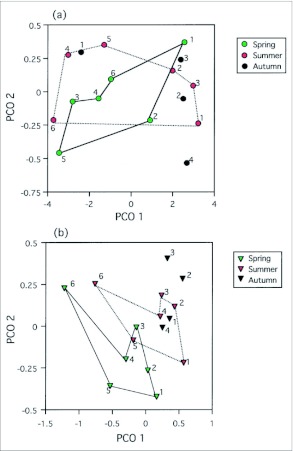
Principal coordinate analysis (PCO) showing the similarities among seasonal populations of (a) *T. quercicola* and (b) *F. yessensis*. PCO axis 1 explains 95.7% and 83.2% of the contribution and PCO axis 2 explains 4.2% and 12.7% for *T. quercicola* and *F. yessensis*, respectively. The numbers in the graphs indicate individual trees. Convex hulls were drawn to compare the relative similarities among six populations between spring and summer.

PCO analysis showed that in spite of a drastic change of genetic difference in *F. yessensis* between spring and summer, the relative position among the six populations was maintained from spring to summer (Figure 3b). In contrast, the pattern of genetic differentiation in *T. quercicola* changed greatly between spring and summer (Figure 3a).

**Figure 4.**
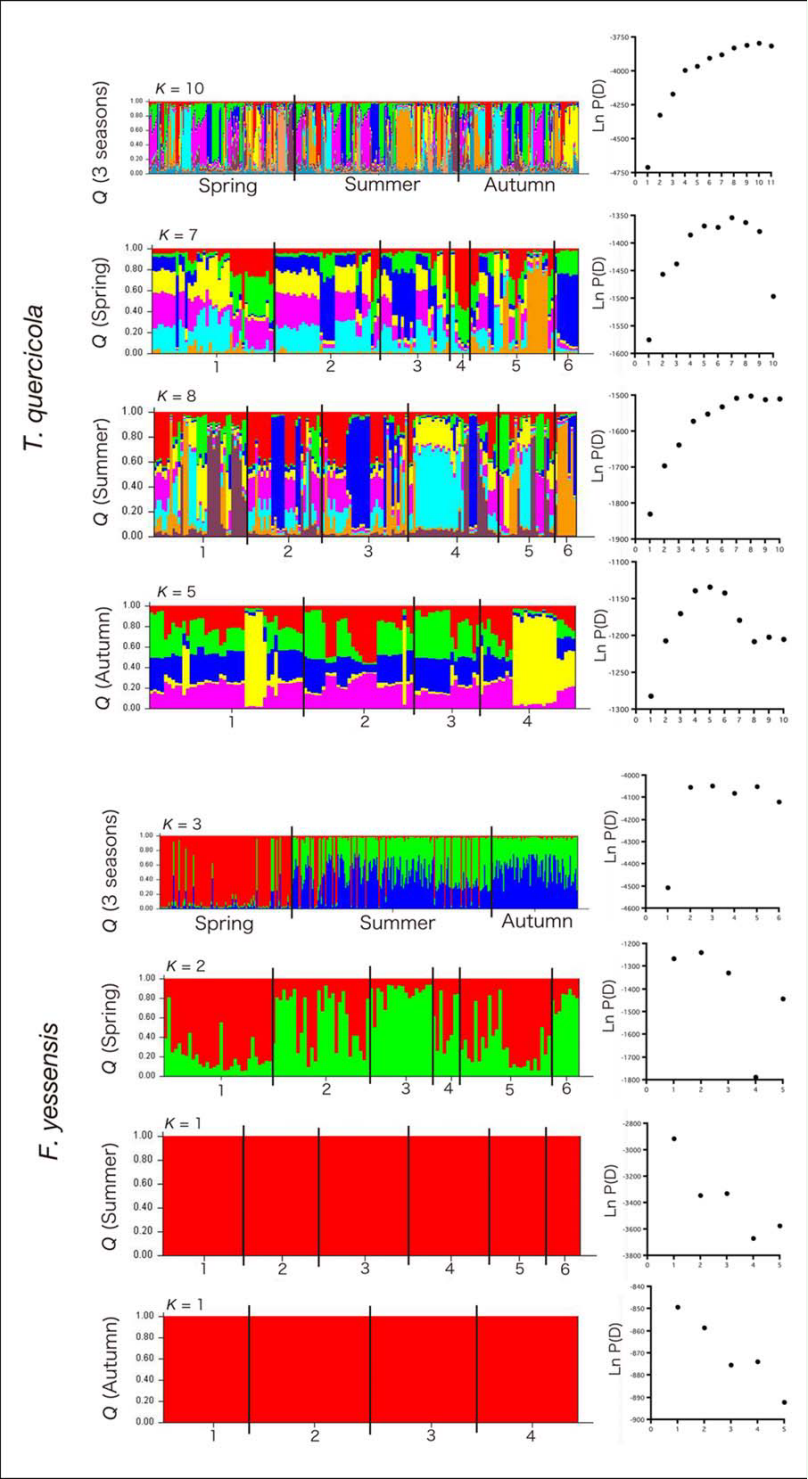
Summary plots of estimates of *Q* for *T. quercicolo* and *F. yessensis* calculated with the entire data set and with the data divided into each season. *Q* indicates the estimated membership coefficients for each individual in each cluster. Each individual is represented by a single vertical line broken into *K* colored segments with lengths proportional to each of the *K* inferred clusters. The graph on the right indicates Bayesian posterior probabilities estimated for *K*. Higher values represent a higher probability of the assumed partition.

The results of Structure analysis for the entire data set including the label of seasons revealed that the number of cryptic populations (*K*) were 10 for *T. quercicola* and three for *F. yessensis* (Figure 4). Clear segmentation between the spring and summer populations was found in *F. yessensis*, but not in *T. quercicola* (Figure 4). Analyses for the sample belonging to each season showed that five to eight clustering and one to two clustering existed in the population of *T. quercicola* and *F. yessensis*, respectively.

**Table 3.**
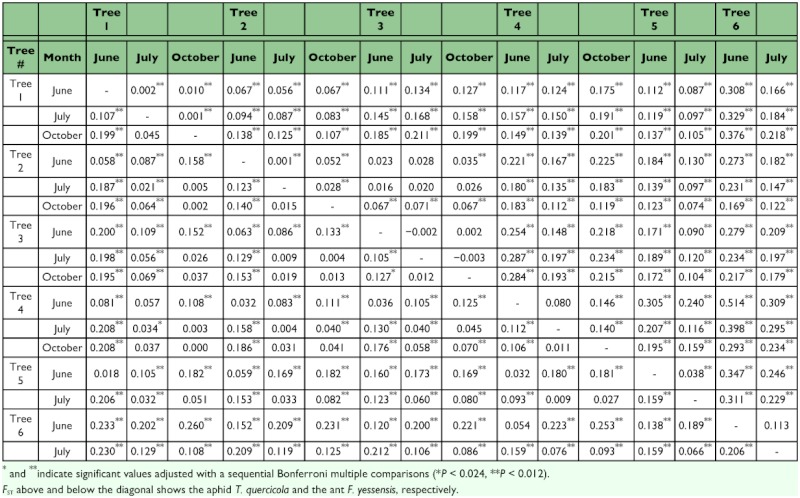
Pairwise *F*_ST_ between host trees in each season.

### Seasonal changes in clonal mixing in *T. quercicola* colonies

**Figure 5.**
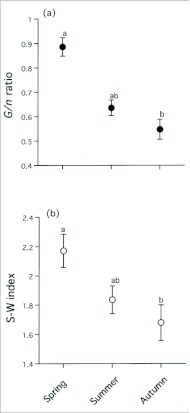
Seasonal changes in the clonal diversity based on (a) *G/n* ratio and (b) S-W index within a *T. quercicola* colony. Mean ± SE. Data points with different letters indicate that a significant difference between seasons was found by multiple comparisons (Tukey's HSD test).

Two-way ANOVA indicated that both the *G/n* ratio and S-W index of *T. quercicola* in each colony decreased with the advance in season (*G/n* ratio, *F*_2, 100_ = 26.92, *P* < 0.0001; S-W index, *F*_2, 100_ = 4.97, *P* = 0.0087). Both the *G/n* ratio and S-W index were high in spring colonies (*G/n* ratio averaging 0.89 ± 0.04 SE; S-W index averaging 2.17 ± 0.11 SE) and low in autumn colonies (*G/n* ratio averaging 0.55 ± 0.04 SE; S-W index averaging 1.68 ± 0.12 SE) (Figures 5a and 5b). Significant effect on both the *G/n* ratio and S-W index was found in trees (*G/n* ratio, *F*_5, 100_ = 6.34, *P* < 0.0001; S-W index, *F*_5, 100_ = 6.23, *P* < 0.0001). The colony in which all *T. quercicola* individuals were assigned to different clones (i.e. a *G/n* ratio = 1) accounted for 67% (22 out of 33 colonies) in spring, 21% (seven of 43 colonies) in summer, and 13% (four of 32 colonies) in autumn (Table 1). Common genotypes that were found through all three seasons were detected only in *T. quercicola* and accounted for 4% (13 of the 317 genotypes).

**Table 4.**
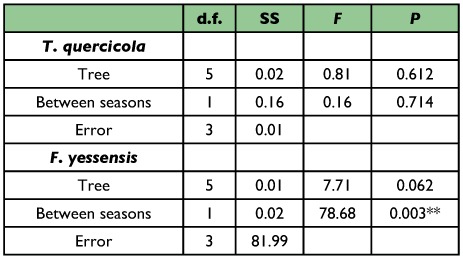
Two-way ANOVA for the effects between seasons on the genetic differentiation in *T. quercicola* and *F. yessensis*. ^**^P < 0.01

## Discussion

### Differences in genetic structure between trees and between seasons

Analysis of microsatellite loci revealed that a significant genetic difference was maintained on the microgeographic scale in both *T. quercicola* and *F. yessensis* populations, and most of the genotypes were found only once on respective host trees throughout the seasons. Microgeographic genetic differences have been reported in a number of studies (De Barro et al. 1995; Sunnucks et al. 1997; Haack et al. 2000; Vorburger 2006), where the authors postulate that stochastic genetic drift or founder effects were mainly responsible for changes in the frequency of genotypes. In the present example, a marked genetic differentiation was evident among the host-associated populations separated by an average of 240m, despite the fact that all *T. quercicola* adults have wings. No isolation by distance, however, was found in *T. quercicola* populations (Figure 2). Moreover, the Structure analysis for *T. quercicola* in each season revealed a close match between genetic partitions and actual trees (Figure 4). Private alleles with a frequency of more than 1% at each locus were found in two loci (Tq15 and Tq23) (Supplemental Data 1). These results suggest that *T. quercicola* has such a low migratory ability that they cannot disperse among the host trees. Attending ants may also inhibit the dispersal of aphids because the aphids are always surrounded by the ants. Besides the limitation of gene flow, selective pressures under heterogeneous host environments would serve to maintain a high level of genetic differences. A number of studies have documented that the performance of herbivores with low migratory ability, such as gall-formers, leafminers, and scale insects, are particularly susceptible to the heterogeneity of their host plants, which sometimes leads to a large amount of genetic variance in insect hatch dates in relation to synchrony with host budburst (Feeny 1970; Akimoto and Yamaguchi 1994; Karban 1989; Komatsu and Akimoto 1995). In the present system, significant differences in honeydew excretion behavior were reported between *T. quercicola* populations on the respective host trees (Yao 2004). This fact supports that *T. quercicola* is affected by heterogeneous environmental conditions.

In contrast to *T. quercicola*, the average of pairwise *F*_ST_ for *F. yessensis* was much lower (Table 3). These results suggest that a weak genetic differentiation exists between trees, and populations of *F. yessensis* have a high relatedness to neighbors, as seen in *Formica paralugubris* type B, which is highly polygynous and has supercolonies (Chapuisat et al. 1997). In *F. paralugubris* B, the number of migrants entering a population per generation was 2.5, which implies that about 99.5 % of the queens are recruited from within the same nest. Chapuisat et al. (1997) suggests that the continuous isolation by distance in the supercolony may be due to nest budding or dispersal of sexual individuals to nearby nests. In the present study, all Structure analyses for *F. yessensis* indicated a partition with the most likely *K* below the actual value of trees (Figure 4). The discrepancy between the numbers of clusters and sampled trees is attributed to differentiation between populations corresponding to the two- dimensional model. Genetic differentiation between *F. yessensis* populations occurred at distance smaller than half of the width of the habitat (see Table 3 and Figure 1), indicating that isolation by distance follows the two-dimensional model (Rousset 1997). Isolation by distance refers to the idea that individuals with slow dispersal may be spatially distributed across a region. In this situation, allele frequencies vary gradually across the region. The underlying Structure model is not well suited for data from this kind of scenario, except for one-dimensional isolation by distance (Pritchard et al. 2000). The inseminated new *F. yessensis* queens have been observed returning to the natal nests after mating (Higashi 1983), supporting that genetic differentiation occurred within close nests and can be maintained by limited gene flow. Thus, newly founded nests will therefore tend to be genetically similar to the nest from which they originated.

A striking, significant genetic difference was found between spring and summer samples in *F. yessensis* but not in *T. quercicola* (Tables 2 and 4, Figures 3 and 4), indicating that changes of genetic composition occurred in *F. yessensis* nests. The demographic pattern of *F. yessensis* was characterized by a sharp increase from late July to late August. Ito (1973) also observed many pupae and new workers in *F. yessensis* nests from mid-June to late September. A large genetic difference in attending workers over summer may therefore be due to eclosion of the first workers and addition of newly-eclosed workers to the foraging force.

It should be noted that even after an alternation of antattended workers, a significant microgeographic genetic difference and isolation by distance were consistently detected among *F. yessensis* populations on the host trees through the seasons (the averages of pairwise *F*_ST_: 0.096 for spring, 0.053 for summer, 0.045 for autumn). Moreover, PCO analysis of *F. yessensis* populations showed that after the addition of newly-eclosed workers, the pattern of genetic similarity between ant populations on the host trees was kept almost constant (Figure 3b). These findings imply that new nests are founded close to natal nests near trees. A number of studies have documented that ant nests often move in response to seasonal shifts in weather (Gordon et al. 2001; Heller and Gordon 2006), disturbances, and changes in food availability (Holway and Case 2000). Furthermore, polydomous colonies that live in more than one nest, bud into many new nests, which may enable colonies to maintain advantageous nutritive, reproductive, or micro-environmental conditions (Banschbach and Herbers 1996, 1999). Cherix (1987) examined the diet composition of *F. yessensis* in our study region and found that the ants depend mainly on aphid honeydew as a food resource. In ant nests experiencing difficulty in foraging new protein resources, inheritance of honeydew resources in the same nest would be advantageous and favored by natural selection. Given that some of the mounds near the study trees where *F. yessensis* workers are actively entering and exiting the nest have been observed not to dislocate their position for nine consecutive years, new nests appear to be founded close to natal nests near trees where honeydew could be available during all seasons. Experimental studies have shown that in *F. paralugubris* B, new queens can mate and stay in their natal nest or seek adoption in a foreign nest after a mating flight (Cherix et al. 1991). If *F. yessensis* queens have a similar philopatric behavior, genetic similarities may be kept in a nest that exploits an aphid population on a host tree as a honeydew resource. It is suggested that the limited distribution of susceptible *Q. dentata* trees enables aphid colonies to persist on a limited number of trees for a long time, resulting in long-lasting mutualistic interactions between *T. quercicola* aphids and the attending ant *F. yessensis* on such trees.

### Seasonal changes in clonal mixing in *T. quercicola*

The present study also showed that a high level of clonal mixing occurred in *T. quercicola* colonies during summer. Both the *G/n* ratio and S-W index of *T. quercicola* within a colony were high in the spring and slowly decreased during summer and into autumn, indicating that aphids of the same genotype gradually increased within a single colony. This is due to an increase by parthenogenesis or selection among clones. Several phenomena, such as high temperatures during summer, a decline in nutritional quality of leaves, and an increase in natural enemies, have been considered as seasonal factors affecting the dynamics of the aphid population (Hales et al. 1997; Karley et at. 2004). In our previous studies, *T. quercicola* populations increased exponentially until early August (Yao 2004), and a following significant reduction was found in both total free amino acid concentration in phloem sap of *Q. dentata* (Yao and Akimoto 2002) and fecundity of aphids (Yao et al. 2000; Yao 2004). If there is interclonal variation, selection may favor some types of clones equipped with a tolerance to high temperatures or high fecundity through interclonal competition. A number of studies demonstrated that under the seasonal selective pressures, a few clone types predominantly increased as the season progressed (Carvalho 1987; Carvalho and Crisp 1987; De Barro et al. 1995; Fuller et al. 1999; Vorburger 2006). In the present study, 13 common clones were found throughout the seasons, but their frequency in autumn (12 %) was lower than reported for other aphid species (Fuller et al. 1999; Vorburger 2006). This may be because of the differences in selective pressures acting on maintenance of polymorphisms between monocultural host plants and heterogeneous long-lived trees.

This study showed that a substantial microgeographic genetic differentiation was found in the aphid *T. quercicola*, while the attending symbiotic ant, *F. yessensis* had a lower genetic variation among the studied trees. As a consequence of limited suitable sites for *T. quercicola*, trees where honeydew was available all year round were occupied by *F. yessensis* over the same period, and the tree could be heritable at the ant colony level. Tracking the seasonal movement of *F. yessensis* workers with a mark-recapture experiment would contribute to a comprehensive understanding of the evolutionary ecology of aphid-ant mutualisms.

## Figures and Tables

**Supplemental Data 1.**
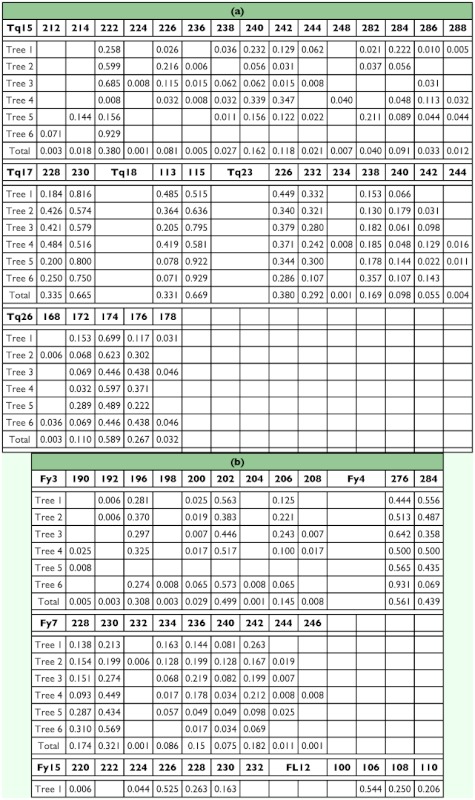
Frequencies of alleles of microsatellite loci for (a) the aphid *Tuberculatus quercicola* and (b) the ant *Formica yessensis*.

**Supplemental Data 2.**
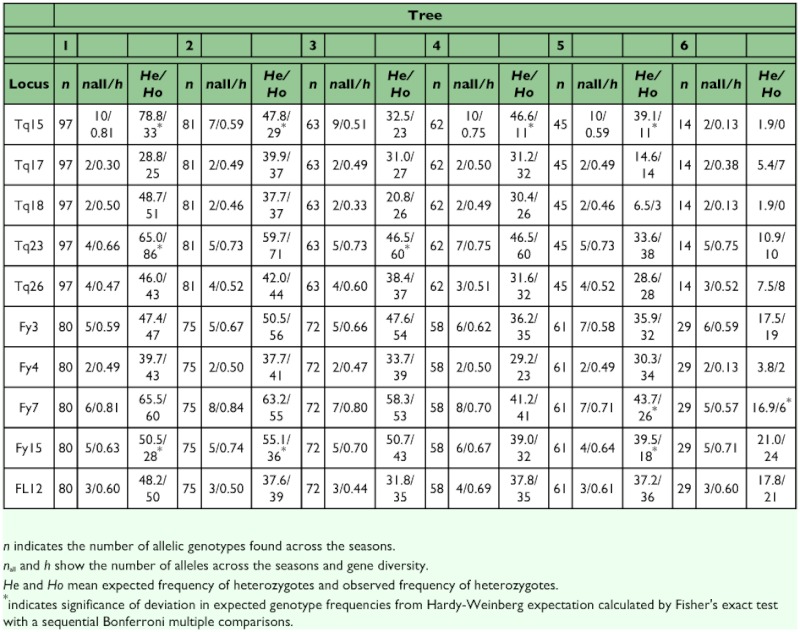
Characteristics of the five *Tuberculatus quercicola* and *Formica yessensis* microsatellite loci in each tree.
